# Mucociliary Clearance Scans Show Infants Undergoing Congenital Cardiac Surgery Have Poor Airway Clearance Function

**DOI:** 10.3389/fcvm.2021.652158

**Published:** 2021-04-23

**Authors:** Phillip S. Adams, Timothy E. Corcoran, Jiuann-Huey Lin, Daniel J. Weiner, Joan Sanchez-de-Toledo, Cecilia W. Lo

**Affiliations:** ^1^Division of Pediatric Anesthesiology, Department of Anesthesiology and Perioperative Medicine, University of Pittsburgh School of Medicine, Pittsburgh, PA, United States; ^2^Division of Critical Care Medicine, Department of Medicine, University of Pittsburgh School of Medicine, Pittsburgh, PA, United States; ^3^Division of Pulmonary Medicine, Department of Pediatrics, University of Pittsburgh School of Medicine, Pittsburgh, PA, United States; ^4^Department of Developmental Biology, University of Pittsburgh School of Medicine, Pittsburgh, PA, United States

**Keywords:** nuclear medicine, congenital heart disease, airway clearance, Technetium 99, critical care, pulmonary

## Abstract

**Background:** Infants undergoing congenital cardiac surgery with cardiopulmonary bypass are at high risk for respiratory complications. As impaired airway mucociliary clearance (MCC) can potentially contribute to pulmonary morbidity, our study objective was to measure airway clearance in infants undergoing congenital cardiac surgery and examine correlation with clinical covariables that may impair airway clearance function.

**Materials and Methods:** Airway clearance in infants was measured over 30 min using inhaled nebulized Technetium 99m sulfur colloid administered either via a nasal cannula or the endotracheal tube in intubated infants. This was conducted bedside with a portable gamma camera. No difficulty was encountered in positioning the gamma camera over the patient, and neither the camera nor the MCC scan interfered with routine medical care or caused any adverse events. Patient and perioperative variables were examined relative to the MCC measurements.

**Results:** We prospectively enrolled 57 infants undergoing congenital cardiac surgery and conducted a single MCC scan per patient. MCC data from 42 patients were analyzable, including five pre-operative, 15 (40.5%) in the immediate post-operative period (days 1–2), and 22 (59.5%) were later post-operative (≥3 days). Pre-operative MCC was inversely proportional to days requiring post-operative mechanical ventilation (*p* = 0.006) and non-invasive positive pressure ventilation (*p* = 0.017). MCC was higher at later post-operative days (*p* = 0.002) with immediate post-operative MCC being lower (3%; 0–13%) than either pre-operative (21%; 4–25%) (*p* = 0.091) or later post-operative MCC (18%; 0–29%) (*p* = 0.054). Among the infants with low post-operative MCC, significantly more were pre-mature [5/19 (26%) vs. 0/18 (0%); *p* = 0.046], were intubated [14/19 (75%) vs. only 7/18 (39%); *p* = 0.033] and were receiving higher FiO_2_ (40%, 27–47% vs. 26%, 21–37%; *p* = 0.015).

**Conclusions:** This is the first study to show that infants undergoing congenital cardiac surgery have impaired MCC. MCC appeared lowest in the immediate post-operative period. Worse MCC was associated with pre-maturity, mechanical ventilation, or receiving higher FiO_2_. These findings suggest MCC scans should be further explored for informing clinical decision making to improve post-surgical respiratory outcomes. The possible therapeutic benefit of airway clearance maneuvers for infants with poor MCC function should also be investigated.

## Introduction

Congenital heart disease (CHD) is among the most prevalent structural birth defects with 25% comprising critical CHD requiring surgical intervention within the first year of life (1). Among patients with CHD, respiratory complications are a significant source of morbidity and mortality after congenital cardiac surgery (2–4). Although the causes for increased respiratory complications are unknown, the extensive exposure to anesthetics, sedatives, and hypothermia during cardiopulmonary bypass (CPB) may inhibit mucociliary clearance (MCC) function (5–8). However, as previously MCC was never functionally assessed in infants, let alone infants with CHD, this precluded evidence-based guidance on best practices or potential therapies to reduce post-surgical pulmonary morbidities in CHD infants undergoing congenital cardiac surgeries.

In this pilot study, our objective was to measure airway clearance in infants undergoing congenital cardiac surgery and also examine for possible correlation with clinical covariables that may impair the airway clearance function. The MCC scan was conducted using a nuclear imaging protocol originally developed for measurement of MCC in older patients with cystic fibrosis (9). We recently showed the effective adaptation of this method for bedside measurement of MCC in intubated and non-intubated critically ill CHD infants (10). Given that the present study is the first use of nebulized Technetium 99m sulfur colloid to quantify airway clearance in infants, we were restricted to only a single scan per infant. In the early phases of this study, different timing and application modality were tested for protocol optimization. Our results indicate airway clearance function is significantly impacted in infants undergoing congenital cardiac surgery.

## Materials and Methods

### Study Design and Setting

This prospective cross-sectional study was conducted at UPMC Children's Hospital of Pittsburgh from October 2015 through October 2018 under an approved institutional review board (IRB) protocol (PRO14070343; approved April 20, 2015). All recruitment and testing occurred in the cardiac intensive care unit or the inpatient cardiac step-down unit. This manuscript was prepared in accordance with the Strengthening the Reporting of Observational Studies in Epidemiology (STROBE) guidelines (11).

### Participants

Infants (≤1 year old) with CHD presenting for cardiac surgery with CPB were assessed for inclusion. Exclusion criteria included patients with known tracheal, bronchial, or lung parenchymal abnormality, those requiring extracorporeal membrane oxygenation pre-operatively, emergent cardiac surgery, or those presenting for heart transplantation. Written informed consent was obtained from parents or legal guardians for all study participants prior to any study activities.

### Mucociliary Clearance Scans

All MCC scans were performed bedside without moving the patients. A single MCC scan was performed surrounding each patient's primary cardiac operation in either the pre-operative, immediate post-operative [post-operative days (POD) 1–2], or later post-operative (POD ≥ 3) period. Scans were conducted bedside with a portable gamma camera (Digirad Ergo; Suwanee, GA, USA) positioned approximately 12–15 cm above the patient's chest. The MCC scan protocol used in this study was developed during the early phases of this study and has been described by Corcoran et al. (10). Only one MCC scan was performed on each patient as specified per IRB approval. For each patient, an initial background scan was conducted, followed by delivery of the tracer, and then 30 consecutive, 1-min dynamic scans were recorded. The percentage of clearance was calculated with decay and background correction. For each scan, 2 ml of 220 nm filtered Technetium 99m sulfur colloid (^99m^Tc-SC) in 2 ml normal saline was nebulized using an Aerogen Solo nebulizer (Dangan, Galway, Ireland). For intubated infants, a 5 mCi dose of ^99m^Tc-SC was used, and the nebulized aerosol was introduced via the endotracheal tube. For non-intubated infants, a 2 mCi dose of ^99m^Tc-SC in nebulized aerosol was delivered by nasal cannula. The use of higher dose for endotracheal delivery helped minimize delivery time and, hence, potential disturbance to patient ventilation. The lower dose with the cannula delivery yielded more precise titration of the dose based on delivery time. Of the 57 MCC scans conducted, 15 were excluded due to low quality from high background associated with non-pulmonary aerosol deposition that interfered with visualization of the lung, or the dose delivered to the lungs was inadequate.

### Variables

The percentage of clearance in the MCC scan was measured with decay and background correction performed, and data were normalized based on first frame counts. The 30-min clearance was assessed independently in the right and left lungs and calculated using the average of normalized data from the last four images. We utilized the higher of the two whole lung clearance measurements because low clearance could be an artifact caused by various conditions, such as endotracheal tube orientation limiting assessment of one lung or the other, high deposition directly at the end of the tube, tube intrusion into the measurement zone, or interference from swallowed tracer in the esophagus for those with non-intubated studies.

Other patient-related variables were abstracted from the medical record and were selected based on previous studies suggesting they could affect MCC (temperature, medications, CPB) as well as other variables that could potentially impact MCC (5–8). Several variables at the time of the patient's MCC scan were documented, including age, time relative to surgery, patient temperature, presence of an endotracheal tube, and medications (opioids, dexmedetomidine, neuromuscular blocking drugs) being administered. Additional prescan variables included pre-maturity status and CHD characterization (single- vs. two-ventricle physiology, conotruncal heart defect). Intraoperative variables recorded included CPB duration, use of deep hypothermic circulatory arrest, and percentage of intraoperative time spent hypothermic (below 35°C).

### Statistical Analysis

Our primary objective was to determine the feasibility of performing bedside MCC scans in infants after congenital cardiac surgery. In addition, we were interested in exploring potential associations between MCC and other perioperative variables. Therefore, we initially began by simply plotting clearance levels based on POD via a scatterplot. The Pearson product-moment correlation coefficient was derived to examine this linear relationship. Given our observation of very low MCC in the immediate post-operative period, we subsequently conducted several pre-operative MCC scans. We then categorized the MCC scans as pre-operative, immediate post-operative (POD 1–2), or late post-operative (POD ≥ 3). Given the non-normal distribution of MCC, Kruskal-Wallis testing with Dunn's *post-hoc* pairwise comparisons with Bonferroni correction of the *p-*value was used to compare MCC between these three epochs.

Next, to more closely examine which variables may impact post-operative MCC, we conducted variable comparisons between infants with high (≥10%) vs. low (<10%) post-operative MCC. Because MCC scans have never been conducted in infants or patients with CHD, we chose 10% as our cutoff as this was roughly the overall median clearance for all post-operative scans. These data are presented as count with percentage, mean with standard deviation, or median with interquartile range for non-normally distributed continuous variables. Categorical intergroup comparisons were made using Pearson chi-squared or Fisher exact tests. Student's *t-*test or Wilcoxon rank-sum testing was used for pairwise comparisons. The 95% confidence intervals (CI) for continuous data are presented, and those for medians were derived using methods described by McGill et al. (12). In addition, we compared the post-operative MCC levels between all categorical variables using Wilcoxon rank-sum testing.

Last, we examined clinical covariables associated with the pre-operative MCC scans we conducted. Scatterplots were generated, and Pearson product-moment correlation coefficients were derived for pre-operative MCC and perioperative variables. Multivariable models were not examined given that this was an exploratory analysis of a novel clinical tool and its association with various perioperative variables. Normality of data was examined using Shapiro-Wilk testing and histograms. No data were transformed. A *p*-value of 0.05 was considered statistically significant. Statistical analyses were completed using Stata/SE 15.1 (College Station, TX, USA).

## Results

**Figure 1:**
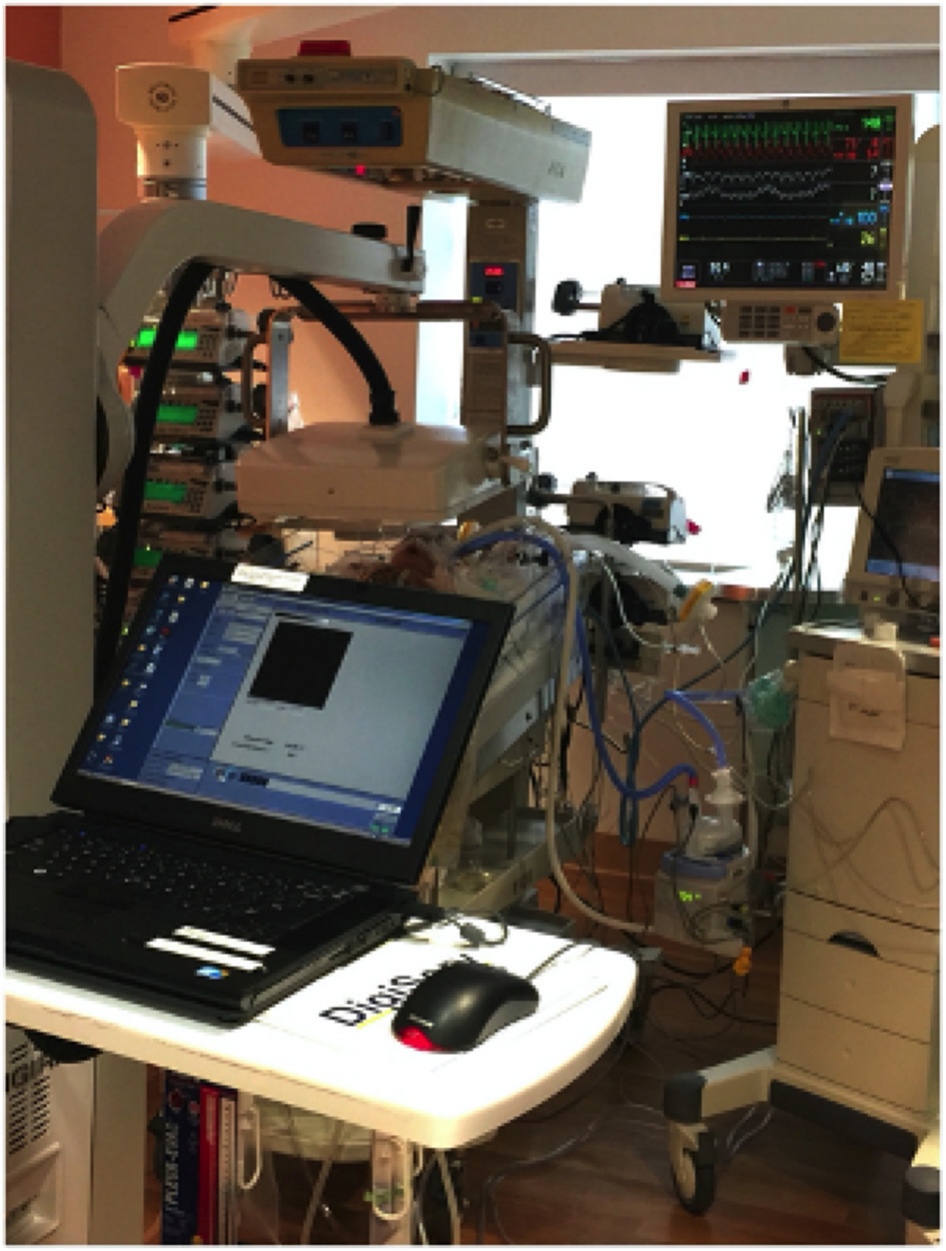
Bedside mucociliary clearance scan for a neonate after congenital cardiac surgery. The Digirad Ergo portable gamma camera is positioned over the patient's chest without touching the patient or interfering with any support devices.

To assess infant MCC after cardiac surgery with CPB, MCC scans were conducted bedside on 57 infants undergoing congenital cardiac surgery. Nebulized ^99m^Tc-SC was delivered into the airway via a nasal canula or endotracheal tube (for intubated infants), and imaging was conducted using a portable gamma camera brought bedside (Supplementary Table 1). There were no issues navigating the camera to the inpatient unit nor in positioning the camera over the patients (Figure 1), and at no time was standard patient care impacted.

Initially, all MCC scans were performed in the immediate post-operative period (POD 1–2). As this revealed consistently low MCC, a small number of pre-operative scans were conducted to exclude possible technical problems with the MCC protocol. Pre-operative scans yielded higher MCC, suggesting the poor MCC in the immediate post-operative period is likely a reflection of the clinical status of the patient. To investigate this further, we also conducted some MCC scans in the later post-operative period (POD ≥ 3).

In total, MCC scans were conducted on 57 patients. Of these scans, 15 scans from mostly the early study period were unreadable due to suboptimal tracer deposition (see section Materials and Methods). Of the remaining 42 readable scans, five were pre-operative and 37 were post-operative. Patient characteristics for the different perioperative epochs (see below) are presented in Supplementary Table 2.

### Pre-operative MCC Scans Show Inverse Correlation to Post-operative Parameters

**Figure 2:**
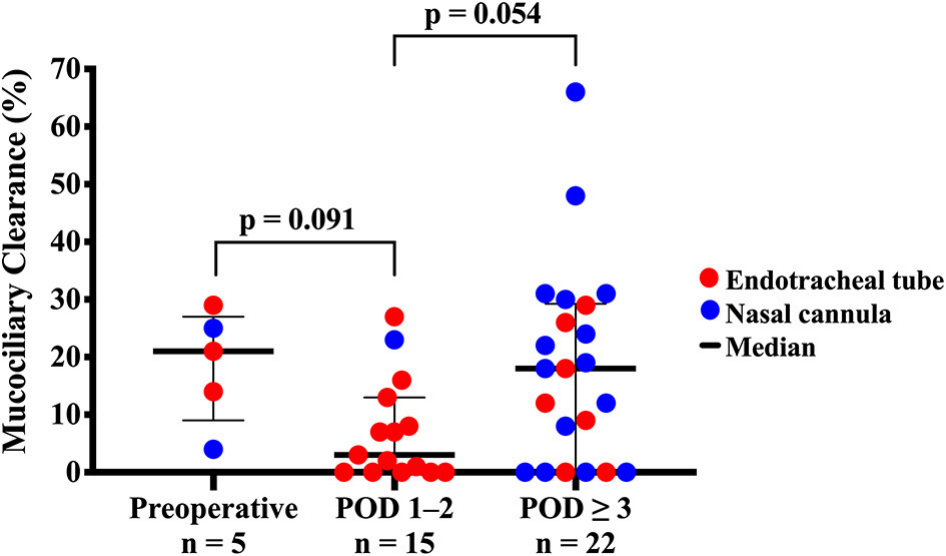
Pre-operative and post-operative mucociliary clearance in infants with congenital heart defects. Infants with congenital heart disease undergoing congenital cardiac surgery had mucociliary clearance measured in one of three different perioperative time points: pre-operative, immediate post-operative (post-operative days 1–2) or later post-operative (≥3 post-operative days). Red dots refer to scans performed via endotracheal tube for intubated infants, and blue dots are scans performed via nasal cannula for non-intubated infants.

**Figure 3:**
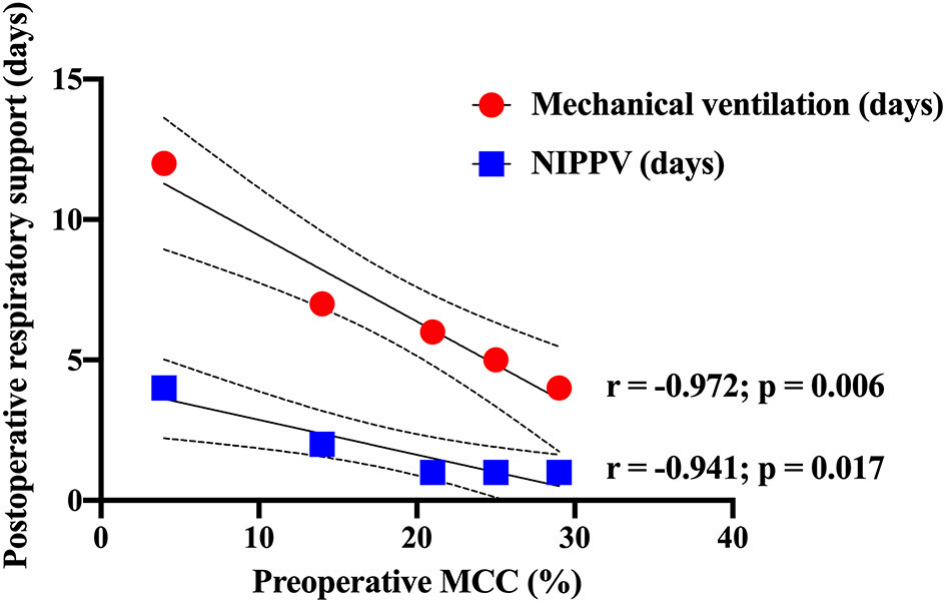
Pre-operative mucociliary clearance associated with post-operative respiratory support. Significant inverse correlations were observed between the percentage of pre-operative mucociliary clearance and post-operative days requiring mechanical ventilation (red dots) and non-invasive positive pressure ventilation (blue squares).

Scans performed on five infants pre-operatively showed robust MCC ≥ 10% in four of the infants (Figure 2). Interestingly, the pre-operative MCC showed a significant inverse correlation with the duration of both post-operative mechanical ventilation days (*r* = −0.972, *p* = 0.006) and days requiring non-invasive positive pressure ventilation (*r* = −0.941, *p* = 0.017) (Figure 3). There were no other significant relationships observed between pre-operative MCC and other perioperative variables (Supplementary Table 3).

### Post-operative MCC Scans Show Reduced Airway Clearance

Post-operative scans were conducted on 37 infants. These were performed at the earliest time post-surgery when the clinical status of the patient was determined to be stable and amenable to the study. Of 37 post-operative scans, 15 (40.5%) were performed in the immediate post-operative period (POD 1–2 days) and 22 (59.5%) in the later post-operative period (POD ≥ 3 days).

**Figure 4:**
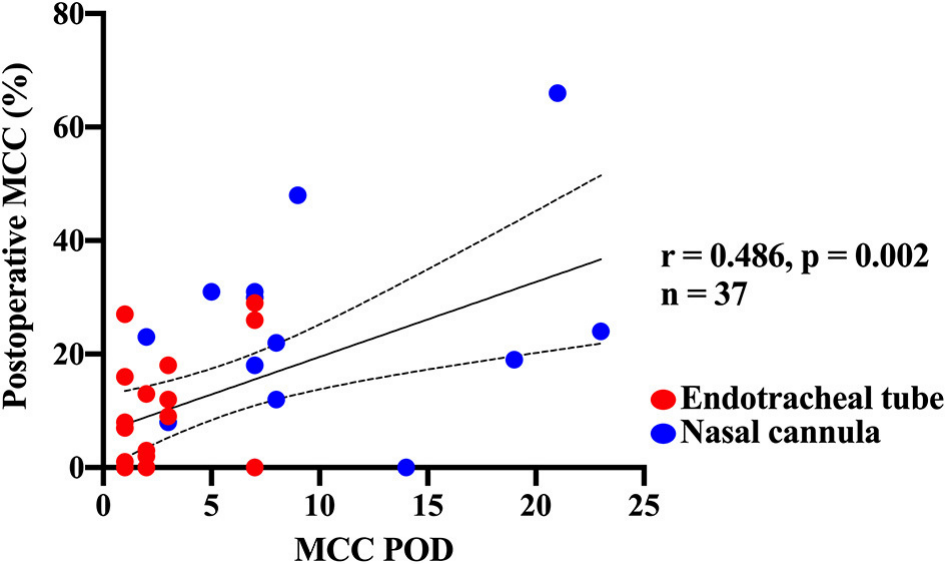
Correlation of post-operative mucociliary clearance function with post-operative day of the scan. A direct correlation was observed between the post-operative day that the mucociliary clearance scan was performed and percentage of mucociliary clearance measured on that day. Red dots denote those who were intubated during their scan, and blue dots indicate those who had nasal cannula studies (non-intubated).

The overall median post-operative MCC was 9% (IQR 0–23%, 95% CI 3–15%). Immediate post-operative MCC (POD 1–2) of 3% (0–13% IQR, 95% CI −2 to 8%) was lower than either the pre-operative group with MCC of 21% (14–25% IQR, 95% CI 13–29%) or the later post-operative group (POD ≥ 3) with MCC of 18% (0–29% IQR, 95% CI 8–28%). However, these differences were not statistically significant (Figure 2). Overall, post-operative MCC showed a significant positive correlation with POD (*p* = 0.002) (Figure 4).

### MCC Correlation With POD, Pre-maturity, Intubation, and Inspired Oxygen

**Figure 5:**
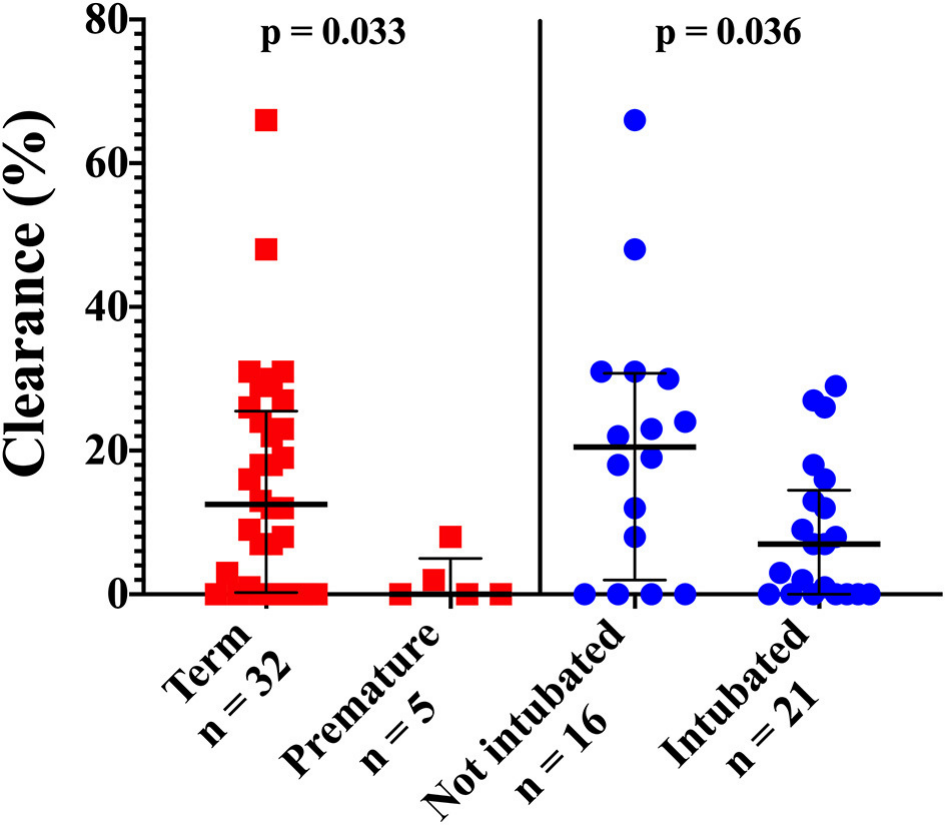
Post-operative variables significantly associated with percentage of mucociliary clearance. Pre-mature infants (born <37 weeks gestational age) and infants who were intubated during their mucociliary clearance scans had significantly lower mucociliary clearance than term patients and those who underwent cannula studies.

Analysis of patient baseline characteristics between those with high (≥10%) vs. low (<10%) post-operative MCC showed significantly more pre-mature infants (<37 weeks gestational age) with low post-operative MCC (Table 1). Infants in the high post-operative MCC group had MCC scans at a later POD, had lower creatinine, were receiving a lower fraction of inspired oxygen, a lower proportion were intubated, and a lower proportion were receiving a neuromuscular blocking drug (Table 1). Post-operative clinical outcomes, such as length of stay and respiratory support, did not differ between the two groups (Table 1). Analysis of the MCC measurements relative to categorical variables showed post-operative MCC was significantly higher in term vs. pre-mature infants, and non-intubated vs. intubated infants (Figure 5). Of the continuous variables, post-operative MCC showed a positive correlation with POD (*r* = 0.486, *p* = 0.002) but a negative correlation with the fraction of inspired oxygen (*r* = −0.408, *p* = 0.012). Supplementary Tables 4, 5 contain the results of other categorical and continuous variables, respectively.

**Table 1:** Comparison of CHD patients with no/low vs. high post-operative mucociliary clearance.

	**0% to** ** <10% clearance**		**≥10% clearance**		***p*-value**
	***n*** = **19**		***n*** = **18**		
	**Value**	**95% CI**	**Value**	**95% CI**	
**Baseline characteristics**					
Male sex, *n* (%)	13 (68%)		9 (50%)		0.254
White non-Hispanic, *n* (%)	16 (84%)		16 (89%)		>0.999
Gestational age, weeks (SD)	37.7 (1.8)	36.8–38.6	38.5 (0.9)	38–39	0.098
Pre-mature, *n* (%)	5 (26%)		0 (0%)		0.046
Single ventricle, *n* (%)	4 (21%)		7 (39%)		0.235
Conotruncal CHD, *n* (%)	11 (58%)		9 (50%)		0.63
**Operative variables**					
Age at surgery, days (IQR)	14 (6–123)	−28 to 56	11 (7–26)	4–18	0.616
Weight at surgery, kg (IQR)	3.3 (2.8–5)	2.5–4.1	3.4 (3.2–4.2)	3–3.8	0.523
STAT category					
2	3 (16%)		2 (11%)		0.086
3	3 (16%)		5 (28%)		
4	11 (58%)		4 (22%)		
5	2 (11%)		7 (39%)		
CPB duration, mins (IQR)	103 (80–144)	80–126	99 (83–117)	86–112	0.773
DHCA, *n* (%)	10 (53%)		9 (50%)		0.873
% operation hypothermic, % (IQR)	53 (29–68)	39–67	51 (29–58)	40–62	0.616
**Variables during MCC scan**					
POD, day (IQR)	2 (1–7)	0–4	7 (3–8)	5–9	0.013
Temperature, °C (SD)	36.4 (0.5)	36.2–36.7	36.5 (0.3)	36.3–36.7	0.533
Creatinine, g/dL (IQR)	0.3 (0.26–0.45)	0.23–0.37	0.23 (0.17–0.3)	0.18–0.28	0.023
FiO_2_, % (IQR)	40 (27–47)	33–47	26 (21–37)	20–32	0.015
Intubated, n (%)	14 (74%)		7 (39%)		0.033
Receiving NMBD, *n* (%)	13 (68%)		6 (33%)		0.033
Receiving opioid, *n* (%)	17 (89%)		13 (72%)		0.232
**Post-operative variables**					
PO LOS, days (IQR)	16 (11-20)	13–19	22 (10-37)	12–32	0.166
CICU LOS, days (IQR)	9 (6–13)	6–12	11 (7–15)	8–12	0.583
Mechanical ventilation, days (IQR)	5 (3–10)	2–8	4 (3–6)	3–5	0.679
NIPPV, days (IQR)	1 (0–2)	0–2	1 (0–5)	−1 to 3	0.649
Respiratory medication used, *n* (%)	7 (37%)		8 (44%)		0.638

*Data presented as count (%), mean (standard deviation), or median (interquartile range)*.

*CHD, congenital heart disease; STAT, Society of Thoracic Surgeons-European Association for Cardio-Thoracic Surgery; CPB, cardiopulmonary bypass; DHCA, deep hypothermic circulatory arrest; POD, post-operative day; FiO_2_, fraction of inspired oxygen; NMBD, neuromuscular blocking drug; PO, post-operative; LOS, length of stay; CICU, cardiac intensive care unit; NIPPV, non-invasive positive pressure ventilation*.

### Older Patients Undergoing Congenital Cardiac Surgery Also Exhibit Low Clearance

The observation of low MCC post-operatively in newborn CHD infants, especially among those that are pre-term, suggests the possibility that the low post-operative MCC in term infants might reflect increased vulnerability of the newborn airways that are not yet fully matured. To investigate this question, we conducted MCC scans on six older CHD patients ranging from 1.7 to 18 years of age also undergoing congenital cardiac surgeries. MCC scans were conducted during POD 1–12 (Supplementary Table 6). This showed similar low post-operative MCC with a 17.7-year-old patient showing zero clearance and four other patients with low post-operative MCC ranging from 1 to 6%. The only patient in this group with high clearance was an 11-year-old female with tetralogy of Fallot who had 34% clearance 3 days post-pulmonary valve replacement (Supplementary Table 6). Overall, these findings show adults undergoing congenital cardiac surgery have low MCC similar to the findings in infants.

## Discussion

Pulmonary complications contribute significantly to post-cardiac surgery morbidity and mortality in infants, but little is known regarding the impact of surgery and cardiopulmonary bypass on functional airway clearance in this patient population. The paucity of data largely reflects the difficulty in working with this critically ill population and the lack of a quantitative test to measure airway clearance function in infants. In this study, we showed bedside MCC scans can be conducted safely in critically ill CHD infants, and this can provide quantitative assessment of airway clearance function. The MCC scan protocol developed provides flexibility to conduct pre-operative or post-operative scans bedside with the in-line ^99m^Tc-SC nebulizer system administered either in intubated patients via the mechanical ventilator or in non-intubated infants using low-flow nasal cannula.

This study, being the first of its kind, by necessity, represents a pilot study that laid the foundation for how MCC scans can be conducted and shows their safety and efficacy for quantitative assessment of airway clearance function in critically ill CHD infants. We made several important observations that may help guide future clinical investigations of infant airway clearance in the post-cardiac surgery time period. Most notably, we observed that MCC was depressed in the earlier post-operative periods. This was significantly correlated with being intubated and receiving neuromuscular blocking drugs (cisatracurium, rocuronium). The physical obstruction from the endotracheal tube combined with the lack of cough from the neuromuscular blockade are likely the factors contributing to low MCC in intubated patients. This suggests earlier extubation may facilitate more rapid recovery of MCC and possibly reduce risk of respiratory complications. Also notable from our study was the finding that all the pre-mature infants (born prior to 37 weeks gestational age) had low post-operative MCC, suggesting pre-maturity poses significant respiratory vulnerability for impaired post-operative MCC. This is support by prior studies showing impaired respiratory dynamics in pre-term infants (13).

The finding of lower supplemental oxygen (FiO_2_) administered in patients with higher post-operative MCC was unexpected. This might reflect the known effects of hyperoxia in causing airway cilia denudation (14). Thus, lower FiO_2_ may enhance preservation of respiratory cilia required for airway clearance function. We note the current neonatal intensive care guideline is to maintain SpO_2_ at 90–95% to minimize oxidative damage. This may have the added benefit of improving airway clearance function by preserving cilia in the airway (15). The finding of higher creatinine levels with low post-operative MCC may reflect pulmonary edema from impaired fluid clearance from post-operative acute kidney injury. Also of significant interest is the observation that pre-operative MCC was inversely correlated with the duration of post-operative respiratory support, findings that require a replication study with a larger cohort.

### Clinical Implications

The observed negative impact of intubation on airway clearance is of significant interest given neonates and infants with low MCC are likely to have increased risk for atelectasis and intrapulmonary shunting from accumulated airway secretions and debris. In these infants, respiratory complications can cause poor post-surgical outcomes even in the setting of a perfect cardiac operation, suggesting the importance of earlier extubations. After congenital cardiac surgery, an extubation failure rate of 6–17% is observed with respiratory dysfunction being the most common reason for extubation failure (16, 17). Extubation failure is associated with worse clinical outcomes, including risk for in-hospital mortality (16, 18). This is observed with 18 times higher odds in those with airway diseases (19). If MCC scans could be used to identify patients with poor airway clearance function, this may perhaps provide earlier opportunity for interventions to improve airway clearance and support earlier extubation.

Our preliminary findings show that those with low pre-operative MCC may be at increased risk for increased post-surgical respiratory complications. Being able to identify patients at risk for low MCC would allow the implementation of both pre- and post-operative therapeutic strategies to promote MCC and improve post-surgical respiratory outcome. Thus, patients identified with low MCC could be treated with mucoactive medications (expectorants, mucolytic agents, mucoregulatory anticholinergics, such as ipratropium bromide) (20). We previously showed ipratropium use is significantly correlated with CHD patients with airway ciliary dysfunction, and Dornase use showed a trend (21). Another therapeutic option is continuous high-frequency oscillations, similar to intrapulmonary percussive ventilation to simulate cough in muscle-relaxed, intubated children. Also possible is delivery of high-flow mini-bursts of air during regular mechanical ventilation to enhance secretion clearance and prevent atelectasis (22). The therapeutic efficacy of these different treatments could be directly assessed using MCC scans to measure airway clearance function.

### Limitations

This study is limited by the relatively small sample size. Additionally, we did not recruit a control group of the same size and age as our study design is that of an observational, cross-sectional study and not a randomized control trial or case-control study. Due to the critical clinical status of CHD infants during the post-operative period, we were unable to obtain MCC scans at the same time point in all patients. Also, a limitation was the fact that only a single MCC scan was conducted per infant. Given the novel nature of infant bedside MCC scans, the IRB allowed only a single MCC scan. With evidence from this study of the efficacy of these scans and the protocol developed that requires only a very low ^99m^Tc-SC dose, this would support future longitudinal studies with multiple pre- and post-operative scans of the same patient. Given that we previously showed respiratory complications are significantly increased in CHD patients with ciliary dysfunction (21), future studies are warranted to investigate whether ciliary dysfunction may contribute to the post-surgical impairment of MCC.

### Conclusion

Our study shows the feasibility to quantify functional airway clearance in infants undergoing cardiac surgery. We observed impairment of airway clearance especially in the early post-operative period. Pre-maturity and remaining intubated were significant risk factors for poor airway clearance. These and other findings suggest post-operative MCC may be improved by early cessation of neuromuscular blocking agents, early extubation, and limiting FiO_2_. Our findings also indicated pre-operative MCC may prognosticate post-surgical respiratory complications, findings that require validation in a larger cohort. As respiratory complications comprise a significant cause of morbidity and mortality after congenital cardiac surgery, insights into the impact of MCC function on post-surgical respiratory outcomes can help formulate evidence-based clinical management strategies to promote airway clearance function and improve the respiratory outcomes of this highly vulnerable CHD infant population.

## Data Availability Statement

The raw data supporting the conclusions of this article will be made available by the authors, without undue reservation.

## Ethics Statement

The studies involving human participants were reviewed and approved by University of Pittsburgh Institutional Review Board (IRB) (PRO14070343; approved April 20, 2015). Written informed consent to participate in this study was provided by the participants' legal guardian/next of kin.

## Author Contributions

PA patient recruitment, study procedures, maintained database, data analysis, drafted original manuscript, edited manuscript, approved final manuscript. TC study procedures, maintained database, data interpretation, edited manuscript, approved final manuscript. J-HL patient recruitment, study procedures, edited manuscript, approved final manuscript. DW study procedures, data interpretation, edited manuscript, approved final manuscript. JS study design, patient recruitment, study procedures, data interpretation, edited manuscript, approved final manuscript. CL study design, obtained funding, data interpretation, edited manuscript, approved final manuscript. All authors contributed to the article and approved the submitted version.

## Conflict of Interest

The authors declare that the research was conducted in the absence of any commercial or financial relationships that could be construed as a potential conflict of interest.
