# Supplementary material for: Mucociliary Clearance Scans Show Infants Undergoing Congenital Cardiac Surgery Have Poor Airway Clearance Function
Source: Front Cardiovasc Med. 2021 Apr 23;8:652158. doi: 10.3389/fcvm.2021.652158 (PMC8102682; doi:10.3389/fcvm.2021.652158)
Supplement: Supplementary file 1 [file Data_Sheet_1.docx]

**SUPPLEMENTAL METHODS**

**Systolic Ventricular Function**

Each patient’s transthoracic echocardiogram immediately prior to surgery (preoperative) and immediately prior to discharge (postoperative) were reviewed. Systemic ventricular systolic function was graded in a blinded fashion by pediatric cardiologists experienced in transthoracic echocardiogram interpretation. Qualitative ventricular function was documented as normal, low normal, mildly depressed, moderately depressed, or severely depressed. Patients were characterized either as normal systolic ventricular function if their echocardiogram was read as normal or as abnormal systolic ventricular function if their echocardiogram was read as low normal, mildly, moderately, or severely depressed.

**Additional Perioperative Variables**

Gestational age was abstract from initial birth histories. Patients were categorized as premature if they were born earlier than 37 weeks’ gestation. All intraoperative temperatures were abstracted from the anesthesia records. The number of values < 35 °C were divided by the total number of temperature values to yield the percent of the operation the patient spent hypothermic. Patients were categorized as having received a muscle-relaxant medication at the time of their mucociliary clearance (MCC) scan if they had received cisatracurium and/or rocuronium on the day of their MCC scan. Patients were categorized as having received an opioid at the time of their MCC scan if there were on an infusion of either fentanyl or morphine, were receiving enteral oxycodone, and/or were receiving intravenous or enteral methadone. Patients were categorized as having received a respiratory medication if at any time during their postoperative recovery they received any of albuterol, N-acetylcysteine, dornase-alpha, ipratropium, or racemic epinephrine. Bacterial infections were defined as a positive bacterial culture obtained at any point in the patient’s postoperative recovery or any documented full antibiotic treatment course for culture-negative sepsis. Viral infections were defined as any positive viral panel obtained at any point in the patient’s postoperative recovery. Patients were categorized as having any postoperative infection if they had either or both a bacterial or viral infection during their recovery.

**SUPPLEMENTAL RESULTS**

| **Supplemental Table 1. List of patients** | | | | | |
| --- | --- | --- | --- | --- | --- |
| **Subject ID** | **CHD Diagnosis** | **% MCC** | **Delivery Method** | **Quality** | **MCC POD** |
| 7578 | HAA, ASD, VSD | 0 | Intubated | Good | 1 |
| 7579 | d-TGA, VSD, HAA | 7 | Intubated | Good | 1 |
| 7585 | HAA, ASD, VSD | 12 | Intubated | Good | 3 |
| 7586 | HLHS | 8 | Intubated | Good | 1 |
| 7593 | d-TGA | 0 | Intubated | Good | 2 |
| 7601 | d-TGA, VSD, PS | 1 | Intubated | Good | 1 |
| 7602 | Ebstein Malformation | 3 | Intubated | Good | 2 |
| 7610 | DORV, TGA, IAA, VSD | 29 | Intubated | Good | -7 |
| 7612 | d-TGA | 14 | Intubated | Good | -22 |
| 7619 | DORV, TGA, HAA, VSD, ASD | 21 | Intubated | Good | -13 |
| 7627 | HAA, BAV, AS | 7 | Intubated | Good | 1 |
| 7633 | DORV, HLV, MA | 9 | Intubated | Good | 3 |
| 7644 | DORV, TGA, HAA | 2 | Intubated | Good | 2 |
| 7645 | HAA, BAV | 0 | Intubated | Good | 1 |
| 7669 | d-TGA | 31 | Cannula | Good | 5 |
| 7689 | Truncus Arteriosus | 0 | Cannula | Good | 14 |
| 7706 | d-TGA, VSD, Coarctation | 13 | Intubated | Good | 2 |
| 7720 | AVSD, TOF | 31 | Cannula | Good | 7 |
| 7730 | d-TGA | 0 | Cannula | Good | 7 |
| 7733 | Critical AS | 26 | Intubated | Good | 7 |
| 7734 | TOF | 0 | Cannula | Good | 7 |
| 7739 | d-TGA | 48 | Cannula | Good | 9 |
| 7746 | Critical AS | 24 | Cannula | Good | 23 |
| 7747 | Truncus Arteriosus, IAA, VSD | 29 | Intubated | Good | 7 |
| 7751 | d-TGA | 0 | Intubated | Good | 7 |
| 7752 | DORV, HLV, MA | 30 | Cannula | Good | 7 |
| 7756 | HAA | 0 | Intubated | Good | 7 |
| 7757 | Tricuspid Atresia | 0 | Cannula | Good | 7 |
| 7760 | d-TGA | 27 | Intubated | Good | 1 |
| 7768 | DORV, HLV, IAA | 19 | Cannula | Good | 19 |
| 7772 | Truncus Arteriosus | 18 | Intubated | Good | 3 |
| 7603 | HAA | 0 | Intubated | Marginal | 2 |
| 7664 | DORV, PS, VSD | 8 | Cannula | Marginal | 3 |
| 7666 | Ebstein Malformation | 4 | Cannula | Marginal | -3 |
| 7679 | HLHS | 25 | Cannula | Marginal | -1 |
| 7711 | HAA, BAV | 23 | Cannula | Marginal | 2 |
| 7722 | HLHS | 18 | Cannula | Marginal | 7 |
| 7731 | HLHS | 66 | Cannula | Marginal | 21 |
| 7748 | TOF | 16 | Intubated | Marginal | 1 |
| 7758 | AVSD | 22 | Cannula | Marginal | 8 |
| 7780 | d-TGA | 0 | Intubated | Marginal | 1 |
| 7793 | TAPVR | 12 | Cannula | Marginal | 8 |
| 7501 | PA/IVS | 0 | Intubated | Poor | 7 |
| 7630 | HLHS, TAPVR | 0 | Intubated | Poor | -20 |
| 7663 | Tricuspid Atresia | Unreadable | Cannula | Poor | 6 |
| 7670 | DILV, TGA, HAA | 0 | Intubated | Poor | 32 |
| 7673 | HAA, AS, VSD | Unreadable | Cannula | Poor | 19 |
| 7678 | HLHS | Unreadable | Cannula | Poor | 7 |
| 7680 | ccTGA, MA, PA | Unreadable | Cannula | Poor | 4 |
| 7694 | d-TGA | Unreadable | Cannula | Poor | -5 |
| 7701 | VSD | 29 | Intubated | Poor | 5 |
| 7708 | d-TGA, HRV, PA | 37 | Intubated | Poor | 2 |
| 7721 | Severe AS, BAV | Unreadable | Cannula | Poor | 7 |
| 7727 | DILV, DORV, HRV, IAA, VSD | Unreadable | Cannula | Poor | 18 |
| 7732 | Truncus Arteriosus | Unreadable | Cannula | Poor | 7 |
| 7745 | d-TGA, VSD, PS | 14 | Cannula | Poor | 9 |
| 7782 | HAA | 8 | Cannula | Poor | 5 |
| CHD = congenital heart disease; MCC = mucociliary clearance; POD = postoperative day; HAA = hypoplastic aortic arch; VSD = ventricular septal defect; ASD = atrial septal defect; TGA = transposition of the great arteries; HLHS = hypoplastic left heart syndrome; PS = pulmonic stenosis; DORV = double-outlet right ventricle; IAA = interrupted aortic arch; BAV = bicuspid aortic valve; AS = aortic stenosis; HLV = hypoplastic left ventricle; MA = mitral atresia; AVSD = atrioventricular septal defect; TOF = tetralogy of Fallot; TAPVR = totally anomalous pulmonary venous return; PA/IVS = pulmonary atresia with intact ventricular septum; HRV = hypoplastic right ventricle; DILV = double-inlet left ventricle | | | | | |

| **Supplemental Table 2. Baseline patient characteristics compared between the three perioperative groups** | | | | |
| --- | --- | --- | --- | --- |
|  | **Preoperative** | **Immediate Postop POD 1-2** | **Later Postop POD ≥ 3** | **p-value** |
|  | **n = 5** | **n = 15** | **n = 22** |  |
| Age at MCC scan, days (IQR) | 3 (2–6) | 19 (8–52) | 24 (16–75) | 0.042* |
| Gestational age, weeks (SD) | 37.2 (2) | 38.1 (1.5) | 38 (1.5) | 0.698 |
| Prematurity, n (%) | 2 (40%) | 2 (13%) | 3 (14%) | 0.351 |
| Conotruncal CHD, n (%) | 3 (60%) | 8 (53%) | 12 (55%) | >0.999 |
| Single ventricle CHD, n (%) | 1 (20%) | 2 (13%) | 9 (41%) | 0.188 |
| Heterotaxy, n (%) | 0 (0%) | 0 (0%) | 1 (5%) | >0.999 |
| STAT category, n (%) |  |  |  |  |
| 2 | 0 (0%) | 1 (7%) | 4 (18%) | 0.219 |
| 3 | 1 (20%) | 2 (13%) | 6 (27%) |  |
| 4 | 3 (60%) | 10 (67%) | 5 (23%) |  |
| 5 | 1 (20%) | 2 (13%) | 7 (32%) |  |
| Intubated, n (%) | 3 (60%) | 14 (93%) | 7 (32%) | <0.001 |
| Abnormal preoperative systolic ventricular function, n (%) | 0 (0%) | 2 (13%) | 3 (14%) | >0.999 |
| Data presented as count with percentage, mean (standard deviation), or median (interquartile range (IQR)) | | | | |
| *Preoperative patients significantly younger than the later postop patients (p=0·018) | | | | |
| POD = postoperative day; MCC = mucociliary clearance; nNO = nasal nitric oxide; CHD = congenital heart disease; STAT = Society of Thoracic Surgeons-European Association for Cardiothoracic Surgery | | | | |

| **Supplemental Table 3. Correlations between variables at time of mucociliary clearance scan and postoperative outcomes with *preoperative* mucociliary clearance (n = 5)** | | |
| --- | --- | --- |
| **Variable** | **r** | **p-value** |
| **During MCC scan** | | |
| Age | -0.8228 | 0.0871 |
| POD | 0.1032 | 0.8688 |
| Temperature | 0.7017 | 0.1866 |
| Hemoglobin | -0.3395 | 0.5762 |
| Creatinine | 0.7245 | 0.1662 |
| Albumin | 0.3273 | 0.7877 |
| SpO_2_ | 0.5119 | 0.3779 |
| FiO_2_ | 0.4274 | 0.4729 |
| Inhaled NO | none received | |
| Intubated | 0.3794 | 0.5287 |
| Received NMBD | 0.3794 | 0.5287 |
| Received opioid | 0.8274 | 0.0838 |
| Received dexmedetomidine | 0.136 | 0.8274 |
| **Postoperative outcomes** | | |
| PO LOS | 0.0979 | 0.8756 |
| CICU LOS | -0.7837 | 0.1168 |
| Systolic function | all had normal function | |
| Dexmedetomidine infusion days | -0.7388 | 0.1538 |
| Fentanyl infusion days | -0.8427 | 0.0731 |
| Milrinone infusion days | -0.8743 | 0.0525 |
| Nicardipine infusion days | -0.3478 | 0.5662 |
| Received any respiratory medication | -0.8274 | 0.0838 |
| Days received albuterol | -0.8274 | 0.0838 |
| Days received dornase-alpha | none received | |
| Days received ipratropium | none received | |
| Any infection | -0.2607 | 0.6719 |
| Bacterial infection | -0.2607 | 0.6719 |
| Viral infection | no viral infection | |
| Mortality | no mortality | |
| MCC = mucociliary clearance; POD = postoperative day; SpO_2_ = arterial oxygen saturation; FiO_2_ = fraction of inspired oxygen; NO = nitric oxide; NMBD = neuromuscular blocking drug; PO = postoperative; LOS = length of stay; CICU - cardiac intensive care unit | | |

| **Supplemental Table 4. Comparison of postoperative mucociliary clearance between patient characteristics and perioperative categorical variables.** | | |
| --- | --- | --- |
|  | **Postoperative Clearance (%, IQR)** | **p-value** |
| **Pre- and intraoperative variables** | | |
| Female | 13 (0–30) | 0.571 |
| Male | 8 (1–19) |  |
| Non-white | 8 (1–22) | 0.988 |
| White, non-Hispanic | 11 (0–24) |  |
| Term | 13 (1–25) | 0.033 |
| Premature | 0 (0–2) |  |
| Biventricular | 5 (0–22) | 0.079 |
| Single ventricle | 18 (8–26) |  |
| Non-conotruncal CHD | 12 (0–23) | 0.921 |
| Conotruncal CHD | 9 (0–23) |  |
| Abnormal preoperative systolic function | 12 (12–18) | 0.489 |
| Normal preoperative systolic function | 8 (0–25) |  |
| No intraoperative DHCA | 10 (0–27) | 0.97 |
| DHCA | 9 (0–23) |  |
| **Variables During MCC Scan** | | |
| Not intubated during MCC | 21 (4–31) | 0.036 |
| Intubated during MCC | 7 (0–13) |  |
| No inhaled NO | 12 (0–24) | 0.184 |
| Receiving inhaled NO | 2 (0–13) |  |
| No NMBD | 19 (0–30) | 0.065 |
| Receiving NMBD | 7 (0–13) |  |
| No opioids | 22 (0–31) | 0.211 |
| Receiving opioids | 8 (0–19) |  |
| No dexmedetomidine | 12 (0–30) | 0.762 |
| Receiving dexmedetomidine | 9 (1–18) |  |
| **Postoperative Variables** | | |
| Abnormal postoperative systolic function | 21 (8–26) | 0.291 |
| Normal postoperative systolic function | 8 (0–22) |  |
| No respiratory medications | 9 (2–19) | 0.882 |
| Received respiratory medications | 16 (0–29) |  |
| No infection | 8 (0–25) | 0.856 |
| Any postoperative infection | 13 (0–18) |  |
| No bacterial infection | 8 (0–24) | 0.629 |
| Any postoperative bacterial infection | 11 (0–17) |  |
| CHD = congenital heart disease; nNO = nasal nitric oxide; DHCA = deep hypothermic circulatory arrest; MCC = mucociliary clearance; NO = nitric oxide; NMBD = neuromuscular blocking drug | | |

| **Supplemental Table 5. Correlations between perioperative continuous variables and postoperative mucociliary clearance** | | |
| --- | --- | --- |
|  | **r** | **p-value** |
| **Patient and perioperative variables** | | |
| Gestational age | 0.2763 | 0.0978 |
| Age at surgery | -0.2084 | 0.2158 |
| Weight at surgery | 0.0249 | 0.8835 |
| CPB duration | -0.1677 | 0.321 |
| Percent of operative hypothermic (< 35° C) | -0.0583 | 0.7316 |
| **Variables at the time of MCC scan** | | |
| Age | -0.1662 | 0.3255 |
| Postoperative day | 0.4862 | 0.0023 |
| Patient temperature | 0.1678 | 0.3207 |
| Hemoglobin | -0.2171 | 0.1968 |
| Creatinine | -0.2657 | 0.1119 |
| Albumin level | 0.129 | 0.4744 |
| SpO_2_ | -0.2661 | 0.1114 |
| FiO_2_ | -0.4077 | 0.0123 |
| CPB = cardiopulmonary bypass; MCC = mucociliary clearance | | |

| **Supplemental Table 6. Toddler, older children, and adolescent postoperative mucociliary clearance scans** | | | | |
| --- | --- | --- | --- | --- |
| **Subject ID** | **Postoperative Day** | **Age (years)** | **% MCC** | **Intubated** |
| 7665 | 1 | 1.7 | 5 | No |
| 7662 | 1 | 17.7 | 0 | No |
| 7360 | 1 | 18.0 | 2 | No |
| 7582 | 2 | 3.1 | 6 | No |
| 7652 | 3 | 11.0 | 34 | No |
| 7185 | 12 | 15.2 | 1 | No |
